# Hemodialysis-Related Pericarditis With Cardiac Tamponade

**DOI:** 10.7759/cureus.24748

**Published:** 2022-05-05

**Authors:** Hirara Watase, Kohei Oka, Fumiko Yamane, Chiaki Sano, Ryuichi Ohta

**Affiliations:** 1 Family Medicine, Shimane Medical University, Izumo, JPN; 2 Community Care, Unnan City Hospital, Unnan, JPN; 3 Community Medicine Management, Shimane University Faculty of Medicine, Izumo, JPN

**Keywords:** end-stage renal disease, japan, cardiac tamponade, rural hospital, hemodialysis related, pericarditis

## Abstract

Pericarditis can cause chest symptoms in dialysis patients. Moreover, it tends to present with various symptoms other than chest pain in patients with end-stage renal disease (ESRD) than in non-ESRD patients. Here, we present the case of an 86-year-old man on maintenance dialysis who was admitted to the hospital with chest discomfort and dyspnea, which led to cardiac tamponade due to unexplained pericardial effusion. The patient underwent pericardial drainage with an epigastric approach. Based on his medical history and pericardial fluid examination, his condition was diagnosed as dialysis-related pericarditis. Non-steroidal anti-inflammatory drugs and prednisolone administration improved the patient’s condition. There are various causes of pericarditis in patients undergoing hemodialysis. It is crucial to examine the patient’s clinical presentation and pericardial fluid volume to clarify the cause of the disease.

## Introduction

Chest symptoms in patients on dialysis can be secondary to acute coronary syndrome, catheter-related infections, pneumonia, pericardial effusions, and various other conditions and reactions to dialysis and drugs administered during dialysis [1]. Pericarditis is observed in 2-21% of patients on dialysis [2]. The commonest symptom of non-end-stage renal disease (ESRD)-associated pericarditis is acute-onset chest pain [3]. However, widespread ST-segment elevation is seen on an electrocardiogram (ECG) in pericarditis compared to typical ST-segment elevation in myocardial infarction and marked ST reciprocity in ischemia [3]. However, pericarditis in patients with ESRD on dialysis is associated with less frequent chest pain (up to 30% of patients are asymptomatic) because weaker myocardial inflammation may not result in ECG abnormalities [2]. A previous study found that only 1-10% of patients on dialysis demonstrate classical ST-segment elevation [3].

Interestingly, the presentation of pericarditis tends to be more diverse in patients with ESRD than that in patients without ESRD. For example, pericarditis in patients with ESRD could present with massive pericardial effusion without symptoms in contrast to pericarditis in patients without ESRD [4]. Patients with ESRD with pericarditis may have a fever, chills, dyspnea, cough, fatigue, chest pain, and elevated body temperature less frequently than patients without ESRD. Additionally, they may develop other signs and symptoms, such as hypotension and heart failure during hemodialysis [4]. Pericardial effusion could also cause these symptoms gradually. A possible cause of pericardial effusion in patients on dialysis is dialysis-related pericarditis. However, not all pericardial effusions are due to inflammation. Chronic pericardial effusions occur more frequently in patients on dialysis because of volume overload [5]. Here, we present the case of an 86-year-old man who was diagnosed with dialysis-related pericarditis with massive pericardial effusion resulting in cardiac tamponade. Pericardial drainage was performed to improve the symptoms and investigate the etiology. Through this case, we report an atypical presentation of dialysis-related pericarditis and evaluate the disease effectively to avoid cardiac tamponade and pericardiocentesis.

## Case presentation

**Figure 1:**
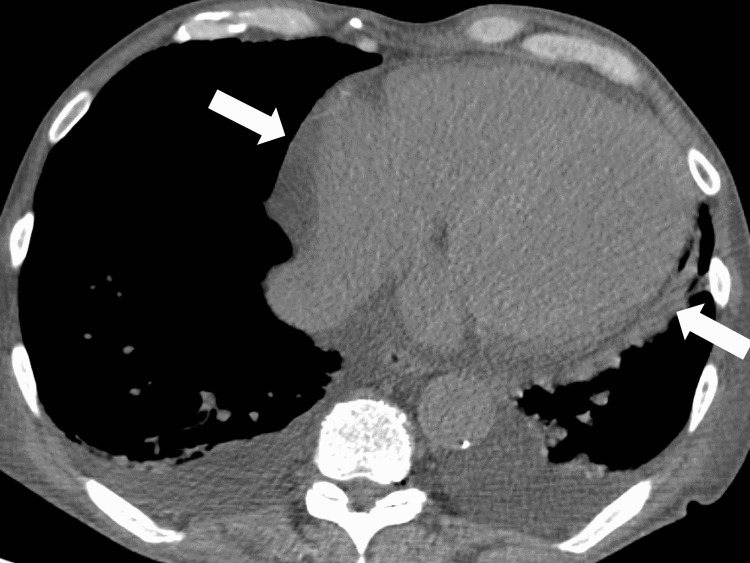
Pericardial effusion on the initial computed tomography

The patient was an 86-year-old man on maintenance dialysis three times a week for chronic renal failure. Dialysis was started two years ago due to chronic renal failure secondary to hypertension. Additionally, the patient had hyperuricemia. He visited our hospital for chest discomfort and dyspnea and was hospitalized for suspected heart failure and cardiac ischemia. Echocardiography revealed an accumulation of pericardial fluid. Cardiac tamponade was considered unlikely because of a lack of hypotension or tachycardia (Figure 1).

**Figure 2:**
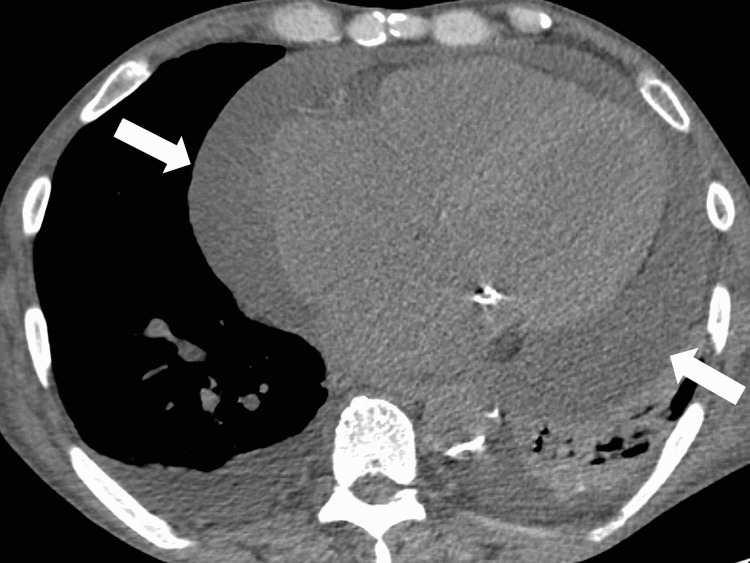
Cardiac tamponade on the subsequent computed tomography

The patient was suspected to have congestive heart failure and was admitted for treatment. The two sets of blood cultures were performed to rule out bacteremia. The patient underwent volume control through dialysis; however, chest pain, respiratory distress, and a fever of 38.6 °C were noted. On the second day of hospitalization, chest computed tomography (CT) revealed a large amount of pericardial fluid (Figure 2).

Two sets of blood cultures were negative, thus reducing the possibility of bacteremia. Consequently, non-steroidal anti-inflammatory drugs (NSAIDs) and colchicine were administered for chest pain and respiratory distress due to suspected hemodialysis-related pericarditis. Additionally, oxygen demand was gradually increasing with a gradually decreasing blood pressure. Furthermore, echocardiography revealed right ventricular collapse, massive pericardial fluid, pendulum-like movement of the heart, and a distended inferior vena cava. Furthermore, laboratory test results revealed high serum levels of C-reactive protein, lactate dehydrogenase, and aspartate aminotransferase (Table 1).

**Table 1:** Laboratory data of the patient. eGFR, estimated glomerular filtration rate; CK, creatine kinase; CRP, C-reactive protein; TSH, thyroid-stimulating hormone; Ig, immunoglobulin; HCV, hepatitis C virus; SARS-CoV-2, severe acute respiratory syndrome coronavirus 2; HIV, human immunodeficiency virus; HBs, hepatitis B surface antigen; HBc, hepatitis B core antigen

Maker	Level	Reference
White blood cells	5900	3.5–9.1 × 10^3^/μL
Neutrophils	78.4	44.0–72.0%
Lymphocytes	16.3	18.0–59.0%
Red blood cells	3.57	3.76–5.50 × 10^6^/μL
Hemoglobin	11.4	11.3–15.2 g/dL
Platelets	17.9	13.0–36.9 × 10^4^/μL
Total protein	6.2	6.5–8.3 g/dL
Albumin	2.7	3.8–5.3 g/dL
Total bilirubin	0.3	0.2–1.2 mg/dl
Aspartate aminotransferase	47	8–38 IU/L
Alanine aminotransferase	36	4–43 IU/L
Alkaline phosphatase	196	106–322 U/L
Lactate dehydrogenase	315	121–245 U/L
Blood urea nitrogen	36.6	8–20 mg/dl
Creatinine	1.12	0.40–1.10 mg/dl
eGFR	35.7	> 60.0 mL/min/L
Serum Na	136	135–150 mEq/L
Serum K	4.5	3.5–5.3 mEq/L
Serum Cl	101	98–110 mEq/L
Serum Ca	8.9	8.8–10.2 mg/dl
Serum P	3.0	2.7–4.6 mg/dl
CK	91	56–244 U/L
CRP	7.78	< 0.30 mg/dl
TSH	10.3	0.35–4.94 μIU/mL
Free T4	1.4	0.70–1.48 ng/dL
Vitamin B1	28	21.3-81.9 pg/mL
Thyroid-stimulating hormone	3.09	0.35–4.94 μIU/mL
Free T4	0.9	0.70–1.48 ng/dL
Folic acid	8.2	> 4.0 ng/mL
Immunoglobin G	1004	870–1700 mg/dl
Immunoglobin M	87	35–220 mg/dl
Immunoglobin A	408	110–410 mg/dl
Immunoglobin E	171	< 173 mg/dl
HBs antigen	0	IU/mL
HBs antibody	0	mIU/mL
HBc antibody	0	S/CO
HCV antibody	0	S/CO
Syphilis treponema antibody	0	S/CO
SARS-CoV-2 antigen	Negative	
Antinuclear antibody	< 40	< 40
Homogeneous	(-)	
Speckled	(-)	
Nucleolar	(-)	
Peripheral	(-)	
Discrete	(-)	
Cytoplasm	(-)	
Proteinase 3-anti-neutrophil cytoplasmic antibody	< 1.0	< 1.0 U/mL
Myeloperoxidase-anti-neutrophil cytoplasmic antibody	< 1.0	< 1.0 U/mL
Anti-SS-A antibody	< 1.0	< 1.0 U/mL
Anti-SS-B antibody	< 1.0	< 1.0 U/mL
Anti-ds-DNA igg antibody	< 10	IU/mL
Rheumatoid factor	0	< 15 U/mL
Anti-citrullinated peptide antibody	0	< 5 U/mL
Beta-D glucan	11	< 20 pg/mL
Interferon-gamma release assays	(-)	

**Figure 3:**
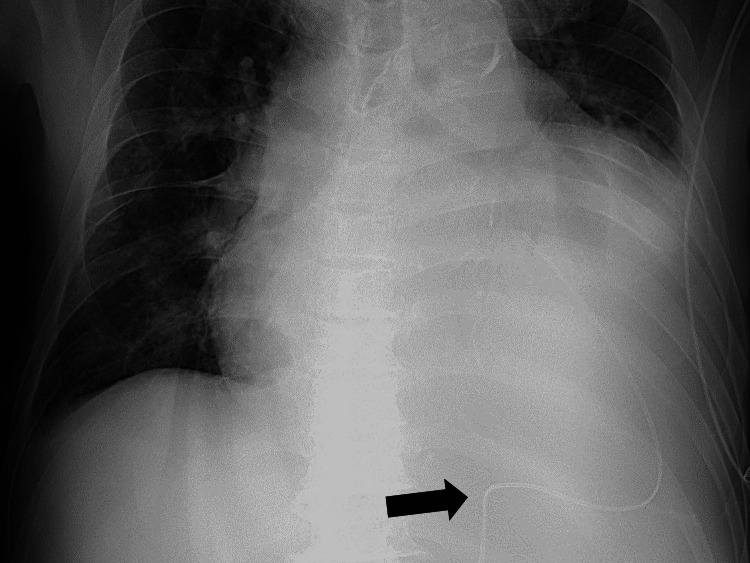
Pericardial drainage catheter for pericardiocentesis

It was determined that the patient had subacute cardiac tamponade. Therefore, a cardiovascular surgeon was consulted for pericardial drainage, which was performed via the epigastric approach using ultrasound (Figure 2). On day 1, approximately 250 mL was drained (Figure 3).

The pericardial fluid was bloody, and no bacteria were noted. Analysis of the pericardial effusion revealed a low pH and lactate level with normal levels of CEA, CA19-9, CYFRA, and adenosine deaminase. The pathological examination did not reveal cells suggestive of malignancy. After approximately 600 mL of bloody fluid was drained in three days, the drain tube was removed after confirming adequate spontaneous drainage. Consequently, oxygen demand gradually decreased, and dyspnea improved. Furthermore, it was determined that oxygen could be terminated five days after the drainage started.

The patient’s fever subsided after the drain was removed. At this point, we suspected dialysis-related, autoimmune (such as rheumatic), or viral pericarditis; therefore, additional blood tests were performed. The results were negative for the following tests: antinuclear antibodies, complement, anti-cyclic citrullinated peptide antibodies, hepatitis B surface antigen, hepatitis C virus antibody, syphilis, β-D glucan, Candida antigen, Aspergillus antigen, mumps, varicella-zoster, simple herpes, Epstein-Barr virus, human parvovirus B19, and human cytomegalovirus immunoglobulin M. Therefore, viral and myocardial infectious pericarditis were considered less likely. Furthermore, bacterial infectious pericarditis was considered not possible due to the subacute course.

Furthermore, cancerous pericarditis was considered less likely because histopathological examination of the pericardial fluid was negative for malignancy. Additionally, the blood tests were negative for tumor markers, such as carcinoembryonic antigen and carbohydrate antigen 19-9, and no apparent tumor lesions were noted on the chest or pelvic CT. Additionally, tests for various autoimmune antibodies, such as antinuclear antibodies were negative; therefore, autoimmune pericarditis was considered unlikely. Consequently, dialysis-related epicardial inflammation was considered the most probable diagnosis. NSAIDs and prednisolone were continued for hemodialysis-resistant hemodialysis-related pericarditis, which resulted in clinical improvements.

## Discussion

We presented the case of a patient on dialysis for chronic renal failure complicated by cardiac tamponade secondary to dialysis-related pericarditis. The probability of developing cardiac tamponade in a patient with pericarditis not on dialysis is 3.1%, in contrast to a rate of approximately 10-20% in a patient on dialysis [6]. Therefore, the probability of cardiac tamponade is exceptionally high in patients on hemodialysis with pericarditis. Additionally, various complications and chest symptoms have been reported in patients on dialysis [7]; therefore, new complaints from patients should be considered seriously.

The cause of cardiac tamponade in patients on hemodialysis should be carefully investigated. Two leading causes of pericarditis in patients on hemodialysis are dialysis-related pericarditis and uremic pericarditis [8]. Uremic pericarditis is defined as pericarditis that occurs before or within eight weeks of initiating dialysis, while dialysis-related pericarditis is defined as pericarditis that occurs in patients who have been on dialysis for >8 weeks [9]. In the current patient, pericardial effusion was observed initially. During the course of time, follow-up of symptoms and CT revealed an exacerbation of pericarditis, which resulted in cardiac tamponade. Chronic pericarditis may have been present in the patient. Therefore, in patients on hemodialysis, cardiac conditions should be followed up closely to detect acute on chronic pericarditis.

Furthermore, given the immunosuppressed state of patients on dialysis, it is critical to consider various pathological conditions as the cause of pericarditis. In the present case, the differential diagnosis included idiopathic, viral, uremic, autoimmune, and neoplastic conditions [10,11]. The pericardial fluid included neutrophils/lymphocytes and no evidence of bacteria. The pericardial fluid findings were suggestive of uremic or dialysis-related pericarditis since both uremic and dialysis-related pericarditis are associated with mononuclear inflammatory cells in the pericardial fluid [12].

The European Society of Cardiology guidelines recommends dialysis, pericardiocentesis/drainage, NSAIDs, corticosteroids, and colchicine to treat pericarditis associated with renal failure [2]. In this patient, removal of toxic substances that were causing pericarditis was attempted with hemodialysis but was stopped due to hypotension. However, the symptoms of right heart failure worsened, and urgent pericardial drainage was performed, which relieved the symptoms. The timing of pericardial drainage was considered due to the progressive pathophysiology of pericardial effusion. As older patients tend to have vague symptoms, their medical help-seeking behavior may be poor [13,14]. Furthermore, in considering the treatment of patients with chronic pericardial effusions, it is crucial to monitor the pericardial fluid volume and the relationship between its rate of increase and clinical symptoms to perform drainage at the appropriate time.

## Conclusions

In this report, we presented the case of cardiac tamponade due to chronic progressive dialysis-related pericarditis in a patient on maintenance hemodialysis. Therefore, pericardial fluid volume and systemic symptoms in patients on dialysis with chronic pericardial effusions should be observed closely. When patients are symptomatic, adjusting rehydration volume and prompt drug therapy may help avoid pericardial drainage and other forms of hemodialysis.
